# Production and carbon footprint of microbial oil from waste lemon peel extract

**DOI:** 10.12688/openreseurope.20856.1

**Published:** 2025-08-28

**Authors:** Vittorio Giorgio Senatore, Essi Paronen, Sofía Martínez-López, Miguel Ayuso, Sofia Ceccarossi, Eveliina Hylkilä, Katri Behm, Mirko Zago, Immacolata Serra, Paola Branduardi

**Affiliations:** 1 Department of Biotechnology and Biosciences, University of Milan-Bicocca, Milan, 20126, Italy; 2 VTT Technical Research Centre of Finland Ltd, Espoo, Uusimaa, 02150, Finland; 3 Environmental Department, National Technological Centre for the Food and Canning Industry, Murcia, 305000, Spain; 4 Department of Earth and Marine Sciences, University of Palermo, Palermo, Sicilia, 90123, Italy; 5 Soft Chemicals S.r.l., Marnate, Lombardia, 21050, Italy

**Keywords:** Cutaneotrichosporon oleaginosum, Microbial oil, Green solvents, Green extraction, Carbon footprint, Impact assessment, Fermentation

## Abstract

**Background:**

The agricultural sector is one of the leading producers of agro-industrial solid organic waste. This waste, mainly disposed of by incineration or landfilled, could be used for the production of high-value chemicals. In this study, a fermentation process for the production of microbial oil from waste lemon extract (LE), an aqueous side-stream deriving from waste lemon peel and pulp processing, was developed and assessed for its impact. Microbial oil can have many diverse applications, from plasticizers in plastic and rubber compounds to moisturizers in cosmetic formulations.

**Methods and results:**

Characterization of LE revealed that its autoclaving process is effective for increasing the concentration of readily available glucose and fructose, reaching 28.77 ± 0.08 g L ^-1^ and 25.68 ± 0.27 g L ^-1^. Nitrogen content was measured too, revealing a C/N ratio of 85, optimal for triggering lipid accumulation in the selected microbial cell factory. Therefore, the oleaginous yeast *Cutaneotrichosporon oleaginosum* was cultivated in an unmodified LE-based medium in 2 L bioreactors, resulting in a lipid accumulation of 0.47 ± 0.08 g _oil_ g _CDW_^-1^. Finally, a new lipid extraction method using green solvents was developed, which allowed to extract and purify 11.29 g of oil, corresponding to 35% of the cell dry weight. The carbon footprint of this laboratory-scale production was estimated to be 71 – 434 kgCO _2_eq kg ^-1^ of microbial oil, with electricity consumption of the fermentation step as the main factor. Simulation of the process in a 300 L fermenter suggests that the overall impact can be drastically reduced with scale-up.

**Conclusions:**

The proposed process is promising in terms of production and does not compete with edible resources and land use. However, the microbial oil yield and the downstream must be optimized to make the process sustainable.

## Plain language summary

This work is part of the results from the European project “Agro2Circular”, which aims at the development of circular economy examples in the European territory by giving new life to the waste of the agricultural sector. In this study, we developed a strategy to convert lemon juice residues into microbial oil. Microbial oil is not made from vegetables (such as olive oil or seed oils), but from microorganisms, such as yeasts, which are similar to the one we use for beer, bread and wine. This microbial oil can be used to substitute vegetable oils in the cosmetic industry, which have a huge environmental impact. To avoid the use of non-renewable chemicals, we also developed a green and safe extraction method. Finally, we performed a carbon footprint analysis, which allowed us to understand the climate impacts and how to further modify the process to improve its sustainability from an environmental point of view.

## Introduction

The agricultural sector is one of the leading producers of agro-industrial solid waste, deriving from crops for food supply, energy production, animal feed, the post-harvest industrial processing, and from the huge amount of plastic and non-recyclable materials vastly employed along the entire value chain. The problem of loss of resources and waste management concerns all industrial fields and has a large impact on both the economy and the environment: each year, about 1.3 billion tons of food are lost or wasted, accounting for $680 billion in industrialized countries and $310 billion in developing countries ( Zhu *et al.*, 2023). Agricultural waste is mainly disposed of by incineration or landfilling, as farmers find these practices to be the most convenient with regard to time, labor, and finance, while also being beneficial for pest control ( Ogbu & Okey, 2023). This waste represents a huge potential resource for the production of high-value chemicals ( Gontard *et al.*, 2018), which can be harvested by cascading (upstream or downstream), with the aim of upgrading the diverse components of the (bio)masses with as much added value as possible, while considering their origin and potential (specific) application(s).

Microorganisms possess the remarkable capacity to metabolize a broad spectrum of substrates and favor the flux of elements and molecules within biogeochemical cycles. This capacity - whether naturally occurring or enhanced via genome editing and metabolic engineering - enables the conversion of diverse feedstocks into an array of novel products. Harnessing this versatility at an industrial scale offers a solution to reduce reliance on fossil-derived resources and to return the biomolecules into biogeochemical cycles in a compatible timeframe, thereby minimizing the environmental impact ( Branduardi, 2021).

In the framework of the Agro2Circular (A2C) project ( https://agro2circular.eu/), with the aim to boost the upcycling of agri-food waste (from fruits and vegetables to multilayer plastic films), we focused our work on the upcycling of lemon peel extract, derived from lemon peel waste from the juicing industry in the region of Murcia (ES). The majority of lemon waste is composed of peels and pulp, which are often discarded or burned; however, these residues contain valuable chemicals, such as volatiles, pigments and essential oils ( Martínez-Abad *et al.*, 2020). In the A2C framework, CTNC (Centro Tecnológico Nacional de la Conserva, Murcia, Spain) extracted polyphenols from this waste, generating an aqueous side-stream enriched in sugars, herein referred to as lemon extract (LE).

LE was used as the substrate to grow the oleaginous yeast *Cutaneotrichosporon oleaginosum*. Under nutrient limitations, but in the presence of abundant organic carbon sources, oleaginous yeasts are able to accumulate large amounts of lipids in the form of storage particles, called droplets or lipid bodies, which can constitute up to 70% (w w ^-1^) of their cell dry weight (CDW) ( Abeln & Chuck, 2021). *C. oleaginosum* is a GRAS (Generally Recognized As Safe) microorganism characterized by fast growth and by the ability to exploit a wide range of carbon sources. As an example, this yeast has already proven to be an excellent candidate for another part of the A2C project, where it was exploited for the upcycling of ethylene glycol derived from the enzymatic hydrolysis of polyethylene terephthalate ( Senatore *et al.*, 2024). The profile of its lipid fraction, characterized by over 50% unsaturated long-chain fatty acids, and composed predominantly of oleic acid and linolenic acid ( Di Fidio *et al.*, 2021; Kourist *et al.*, 2015), can be of interest in cosmetics. For example, argan oil is mainly composed of oleic and linoleic acids, and microbial production might represent an alternative solution to the more traditional plant-based resources, which suffer from many limitations such as seasonality and land use.

With the aim of investigating the potential of LE in the production of microbial oil, in this study we focused on the ability of *C. oleaginosum* to grow and accumulate lipids using this side stream rich in sugars. Moreover, we also proposed an alternative approach for the extraction of lipids from yeast biomass, substituting the traditional potentially carcinogenic Folch protocol (requiring chloroform and methanol) with a combination of two green and safer solvent blends (ASTROBIO™ Green Solvents) chosen from the portfolio of Soft Chemicals S.r.l.

As new bio-based and circular products are developed, such as the microbial oil from agri-food waste in this study, it is necessary to evaluate the environmental impact of the new solutions to assess whether they are more sustainable than other microbial-based solutions used in different applications. Some LCA studies have been published in the field of yeast-based microbial oil applications. These studies examine microbial oils in the use of biofuels ( Chopra *et al.*, 2020; Longati *et al.*, 2022; Sae-ngae *et al.*, 2020 and Sharma *et al.*, 2020) and in water treatment ( Vera *et al.*, 2015). LCA studies that cover only microbial oil production were performed by Abelleira *et al.* (2024), Balina *et al.* (2023), Bonatsos *et al.* (2020) and Masri *et al.* (2019). These studies highlight some critical points of the current microbial oil production process. For instance, Bonatsos *et al.* (2020) reported the high impact of (i) using monosaccharides and yeast extract as nutrient sources, (ii) high water consumption, and (iii) using hexane as solvent for oil extraction ( Bonatsos *et al.*, 2020). Moreover, Abelleira *et al.* reported that biomass dehydration - necessary for efficient oil extraction - also has high energy requirements ( Abelleira *et al.*, 2024). In an effort to understand if our process can overcome the current challenges, this study also included the examination of the carbon footprint of the process.

## Materials and Methods

### Strains and media

The haploid Crabtree-negative yeast *Cutaneotrichosporon oleaginosum* (culture collection ATCC 20509, previously known as *Trichosporon oleaginosum*) was used in the experiments. The strain was maintained in 20% (v v ^-1^) glycerol at - 80 °C after growth in YPD medium composed of (per liter): yeast extract 10 g, peptone 20 g, and glucose 20 g.

### Reagents

Unless otherwise stated, all reagents were obtained from Merck KGaA (Darmstadt, Germany).

### Generation of Lemon Extract (LE) from polyphenol extraction from citrus waste

Verna lemon ( *Citrus limon* (L.) *Burm*. f.) was collected and processed by Citromil, S.L, Spain. After peeling and juice extraction, both peel (after oil extraction) and pulp were considered as citrus waste material for further valorization in A2C by CTNC (Centro Tecnológico Nacional de la Conserva, Murcia, Spain).

The citrus waste from the juicing company was transported to CTNC in a lorry with a conservation container within 30 min. Since the raw material was initially washed meticulously by the company, the citrus residue was directly subjected to a pre-treatment of homogenization (cutting) using a cubing machine (Model G-A, Urschel), reducing the particle size to 1 cm × 1 cm.

For the extraction phase, 50 kg of lemon peel residues were accurately weighed, mixed in a 1:3 ratio with reverse osmosis water, and introduced into an industrial vacuum mixing reactor, to which 5 g of a multi-enzyme cellulase complex were added, and the mixture was agitated at 25 °C for 1 h. Enzyme inactivation was carried out by incubating the sample at 98 °C for 25 min. The samples were cooled to room temperature, phase separation was carried out using a decanter, and both phases underwent specific processing for valorization.

Specifically, the liquid phase was treated for the recovery of polyphenols. For this purpose, it was concentrated using a pilot filtration plant (SIVE Fluid Systems). Then, for the purification of phenolic compounds, adsorption-desorption processes were employed, using resins such as PAD950 resin (Ecolab Purolite™). A flow rate of 80 L h ^-1^ was used and three 15-minute adsorption cycles were carried out, which is the estimated time after which the resin becomes saturated. Once the three cycles were completed, the phenolic compounds were eluted with ethanol 96%, freeze-dried and evaluated.

Total polyphenol content was analyzed according to the Folin-Ciocalteu method. The presence of specific phenolic compounds in the extract was determined by High-Performance Liquid Chromatography (HPLC).

The lemon permeate liquid extract (hereinafter, lemon extract LE) after the adsorption-desorption process was concentrated to 18–20 ºBrix and evaluated as substrate for the production of microbial oil.

### Characterization of sugar and nitrogen content of Lemon Extract

Sugar and nitrogen content of LE as provided by CTNC and after autoclaving (121 °C, 20 min, 2 atm) was measured with commercially available kits from Megazyme: K-FRUGL was used to measure the glucose and fructose content; K-LARGE and K-PANOPA were used to measure the Total Yeast Available Nitrogen (YAN _T_), comprised of free ammonium ions, urea, primary amino nitrogen from amino acids and the guanidine group of L-arginine. Presence of additional compounds was assessed by HPLC analysis.

### Growth conditions in 24 wells microplates

Different LE-based media were prepared for the medium optimization experiment ( Table 1). Autoclaved LE (LE80) was used as the unbalanced medium for oil accumulation; to obtain a balanced growth medium, urea was added to a final concentration of 4.5 g L ^-1^, corresponding to a C/N ratio of 8.8 as in the standard synthetic minimal medium ( Verduyn *et al.*, 1992); this medium was called LE8. Additionally, the effects of trace elements and vitamins (v) and the effects of MgSO _4_·7H _2_O 0.5 g L ^-1^, KH _2_PO _4_ 3 g L ^-1^ (salts, s) were tested either alone or in combination. This resulted in a total of eight different growth media, as described in Table 1. All media were buffered with 100 mM potassium hydrogen phthalate buffer, pH 6.

**Table 1.** Composition of LE-based media in 24 wells microplates. Lemon extract (LE) was autoclaved and buffered with 100 mM potassium hydrogen phthalate buffer, pH 6 (LE80); to reach a C/N of 8.8, urea 4.5 g/L was added (LE8). Both media were modified by adding either vitamins and trace elements (v), salts (s), both (vs) or no additional nutrient. Vitamins and trace elements consisted of (per liter): D-biotin 0.10 mg; calcium D-pantothenate 2 mg; nicotinic acid 2 mg; *myo*-inositol 50 mg, and EDTA, 30 mg; ZnSO _4_·7H _2_O, 9 mg; CoCl _2_·6H _2_O, 0.6 mg; MnCl _2_·4H _2_O, 2 mg; CuSO _4_·5H _2_O, 0.6 mg; CaCl _2_·2H _2_O, 9 mg; FeSO _4_·7H _2_O, 6 mg; Na _2_MoO _4_·2H _2_O, 0.8 mg; H _3_BO _3_, 2 mg; KI, 0.2 mg; thiamine hydrochloride, 2 mg; pyridoxal hydrochloride, 2 mg; *para-*aminobenzoic acid, 0.4 mg. Salts consisted in (per liter): KH _2_PO _4_, 3 g; MgSO _4_·7H _2_O, 0.5 g. Composition of vitamins, trace elements and salts was obtained from ( Verduyn *et al.*, 1992).

C/N	Base	Medium composition	
**80**	Buffered autoclaved LE	LE80	LE
		LE80.v	LE + vitamins and trace elements
		LE80.s	LE + salts
		LE80.vs	LE + vitamins and trace elements + salts
**8.8**	Buffered autoclaved LE + urea 4.5 g L ^-1^	LE8	LE
		LE8.v	LE + vitamins and trace elements
		LE8.s	LE + salts
		LE8.vs	LE + vitamins and trace elements + salts

Seed cultures of *C. oleaginosum* from YPD plates were grown in 50 mL glass tubes filled with 10 mL YPD for 24 h. Cells were then harvested, washed and inoculated in a 24-deepwell microplate (CR1424, manufactured by Enzyscreen, Heemstede, The Netherlands) filled with 2 mL of the different LE-based media under evaluation, at a final OD of 0.1. Growth was performed at 30 °C under constant agitation (250 rpm) in the Growth Profiler 960 (Enzyscreen, Heemstede, The Netherlands). Samples were collected at the end of the experiment (72 h) for HPLC analysis and total lipid evaluation.

### Analytical method for total lipid extraction

Total lipid accumulation in *C. oleaginosum* from the experiment in 24-deepwell microplates was evaluated with the Folch extraction method ( Folch *et al.*, 1957). Cells from 1 mL samples were dried overnight at 60 °C in pre-scaled 1.5 mL tubes. The next day, the tubes were weighed to obtain the total cell dry weight (g L ^-1^). Then, 500 μL of 1:2 MeOH/CHCl _3_ solution were added. The tubes were vortexed, parafilmed and incubated overnight in a thermoblock at 40 °C, 1400 rpm overnight. Afterwards, the samples were centrifuged (5’, 21000 g) and the supernatant was transferred into new, pre-scaled 1.5 mL tubes. The solvent was evaporated for 24 h at 60 °C under vacuum; the dry 1.5 mL tubes were then weighed to obtain the total amount of lipids.

***Process conditions in batch bioreactor***

For batch fermentations, 2 L stirred tank bioreactors (BIOSTAT® A plus, Sartorius Stedim Biotech GmbH, Goettingen, Germany) equipped with Visiferm DO ECS 225 for pO _2_ measurement and Easyferm Plus K8 200 for pH measurement (both from Hamilton Bonaduz AG, Bonaduz, Switzerland) were used at a working volume of 1000 mL. The temperature was kept constant at 30 °C and the pH was set to 6.0, maintained by automatic addition of 2 M KOH and 1 M HCl. The stirring rate was set to 300 rpm in cascade to maintain the oxygen concentration, which was set to 25% saturation to guarantee a completely aerobic condition for the cell culture. Filtered air (pore size 0.2 μm) was continuously sparged through the reactor at a flow rate of 1 vvm. Foam formation was controlled by the addition of polypropylene glycol (PPG) at a concentration of 1 mL/L. Gas analyzers (BlueVery, BlueSens gas sensor GmbH) were attached to the outgas for online measurement of %CO _2_ and %O _2_ in the air.

Seed cultures from YPD plates were grown for 8 h in glass tubes in 10 mL YPD; cells were then inoculated for the intermediate inoculum (starting OD 0.05) in 250 mL baffled shake flasks containing 50 mL of pre-inocula medium and grown overnight (16 h). The pre-inocula were performed in a rotary shaker (stroke: 1.9 cm) at 300 rpm and 30 °C. For the inoculum, cells were harvested, washed with sterile dH _2_O, and used to inoculate the bioreactor (starting OD _600_ = 0.5).

The pre-inocula medium was buffered (autoclaved) LE8.vs, consisting of LE diluted to a total final concentration of glucose and fructose of 45 g L ^-1^, urea 4.5 g L ^-1^, MgSO _4_·7H _2_O 0.5 g L ^-1^, KH _2_PO _4_ 3 g L ^-1^, trace elements 1X and vitamins 1X as described above. The medium was buffered with 100 mM potassium hydrogen phthalate buffer, pH 6. The medium for the production was LE80 medium, consisting of autoclaved LE diluted to a total final concentration of glucose and fructose of 45 g L ^-1^ (roughly 0.8 L for a final volume of 1 L).

***Small scale extraction trials with green solvents***

After the fermentation was stopped (52 h), 12 samples of 1 mL each were collected from each reactor in pre-weighted 1.5 mL tubes to test the extraction methods. Each sample was centrifuged (20 min, 4 °C, 21 000 g) and the supernatant was discarded; the pellets were frozen at -20 °C overnight. Half of the samples were dried overnight at 60 °C. Samples were thawed and weighed to measure the (net) amount of dry or wet biomass. Both dry and wet pellets were then processed in the same way: five different solvent combinations ( Table 2) were compared to the standard Folch extraction; in particular, 1000 μL of solvent were added to each tube and samples were incubated under strong agitation (1400 rpm) at 40 °C for 24 h.

**Table 2.** Extraction solutions tested for lipid extraction. Small scale lipid extractions were performed in 1.5 mL tubes using 1000 μL of solvent. For wet samples, NaCl 0.9% was added at the end of the extraction for phase separation: the required volumes for the creation of a biphasic system are listed in the last column. The extraction with MeOH/CHCl _3_ was adapted from ( Folch *et al.*, 1957); the extraction with EtOH/EtOAc was adapted from ( Breil *et al.*, 2017); the remaining solvents were provided by ASTROBIO™. MeOH: methanol; CHCl _3_: chloroform; EtOH: ethanol; EtOAc: ethyl acetate.

Extraction solution	Ratio	Volume of solvent (μl)	Volume of NaCl 0.9% (μl)
MeOH/CHCl _3_	1:2	1000	200
EtOH/EtOAc	1:2	1000	500
BA/XT	1:2	1000	400
BA/SD	1:2	1000	400
BA/K1	1:2	1000	333
BA/K100	1:2	1000	200

Then, the dried samples were centrifuged, and the supernatant (containing the extracted lipids) was transferred into a new pre-weighted 1.5 mL tube. For the wet samples, NaCl 0.9% was added until the formation of two (or more) distinct phases ( Table 2); samples were centrifuged (5’, 21 000 g), and the organic phase (containing the extracted lipids) was transferred into a new pre-weighted 1.5 mL tube. The organic phase is the lightest for all the solvents used, except for MeOH/CHCl _3_. Solvents were then evaporated under vacuum at 60 °C for 24 h, and weighed to measure the yield of extracted lipids per gram of cell dry weight.

The Folch method was included as the reference condition; ethanol/ethyl acetate was included as a suggested alternative from an in-depth study on lipid extraction from yeasts ( Breil *et al.*, 2017). The remaining solvent blends were kindly provided by ASTROBIO™ Green Solvents Division of Soft Chemicals S.r.l.; the specific tested combinations were determined according to their solvency power and physical-chemical properties of the mixtures and recommended by the company itself.

### Large-scale lipid extraction from wet biomass with BA/K1 1:2

The fermentation broth from both reactors was collected in a 1 L glass graduated cylinder and the overall volume was measured. Then, the broth was transferred in pre-weighed 500 mL centrifuge bottles and was pelleted at 4000 g for 1 h at 4 °C. The volume of the supernatant (spent LE) was measured with a graduated cylinder and then discarded. The remaining pellet was weighed and frozen at -20 °C until oil extraction.

For the large-scale extraction, the frozen biomass was thawed and transferred into a 5 L beaker with 500 mL of BA and 1000 mL of K1; the suspension was stirred for 24h at room temperature to minimize solvent evaporation; about 40 mL g _CDW_^-1^ (or 10 mL g _wet pellet_^-1^) of BA/K1 were used. At the end of the extraction, 600 mL of NaCl 0.9% were added for phase separation, the solution was mixed for 15 min and then transferred to centrifuge bottles to decant. The organic fraction (top layer) was transferred into a pre-weighted 1 L evaporating flask and the solvent was evaporated using a rotavapor (67 °C, 50 rpm).

### Quantification of sugars and citric acid by HPLC

Routine HPLC analyses were performed to quantify the amount of glucose, fructose, sucrose and citric acid. Prior to analysis, all samples were centrifuged (21,000 g, 20 min) and diluted when necessary. The HPLC was equipped with a Rezex ROA-Organic Acid H+ (8%) Ion Exclusion column 300 × 7.8 mm, 8 μm (Phenomenex); 10 μL of samples were injected in the column. The mobile phase was H _2_SO _4_ 0.005 N, at a flow of 0.5 mL min ^-1^; column temperature was set to 40 °C. Separated components were detected by a refractive index detector (RID) and by variable wavelength detector (VWD) set at 210 nm. Peaks were identified by comparison with reference standards dissolved in ultrapure H _2_O (18 MΩ). Calibration curves for peak quantification were prepared in a range between 0.625 and 20 g L ^-1^.

A modified method was used for the initial characterization of sugar content in LE. For these preliminary analyses, the HPLC was equipped with a Rezex RPM-Monosaccharide Pb+2 (8%) Ion Exclusion column 300 × 7.8 mm, 8 μm (Phenomenex); 10 μL of samples were injected in the column. The mobile phase was ultrapure H _2_O (18 MΩ), at a flow of 0.5 mL min ^-1^; column temperature was set to 40 °C. Separated components were detected by a refractive index detector (RID) and by variable wavelength detector (VWD) set at 210 nm. Peaks were identified by comparison with reference standards (glucose, fructose, sucrose, citric acid) dissolved in ultrapure H _2_O (18 MΩ). Calibration curves for peak quantification were prepared in a range between 0.625 and 20 g L ^-1^.

### Carbon footprint calculation

Carbon footprint calculation of microbial oil production was conducted based on the Life Cycle Assessment (LCA) method (ISO 14040:2006, ISO 14044:2006), commonly used for assessing the potential environmental impacts of a product or service. The life cycle of a product (or service) starts from the raw material and energy acquisition, and includes product manufacturing, transportations, and typically also use phase and final disposal at the end of life.

The carbon footprint was modeled with a cradle-to-gate system boundary, excluding the use and end-of-life stages. The characterization factors of Environmental Footprint 3.1 (EF 3.1) impact assessment methodology ( European Commission, 2021) were used in the assessment. The method includes 16 impact categories, but in this study only climate change as an impact category was assessed. The calculations were carried out using Sulca software ( Sulca, 2024).

In LCA, the included life cycle stages are built from processes having inputs (e.g. raw material, energy, other resources) and outputs (e.g. products, waste, emissions), connecting the processes to previous and following ones. The data was collected from project partners with an Excel template and interviews. This primary data collection was supported by academic literature, i.e., secondary data. Secondary data used in the assessment was from ecoinvent 3.10 database, using the system model “cut-off by classification”.

***Goal and scope***

The goal of this study was to evaluate the early-stage carbon footprint of microbial oil manufactured on a laboratory scale from lemon waste. Additionally, the aim was to identify the life cycle stages and process types that contributed the most to the carbon footprint of microbial oil production for future technology development purposes.

***Functional unit***

The functional unit of the calculation was 11.79 g of microbial oil manufactured at IndBiotech Lab at University of Milano Bicocca.

***System boundary***

**Figure 1.**
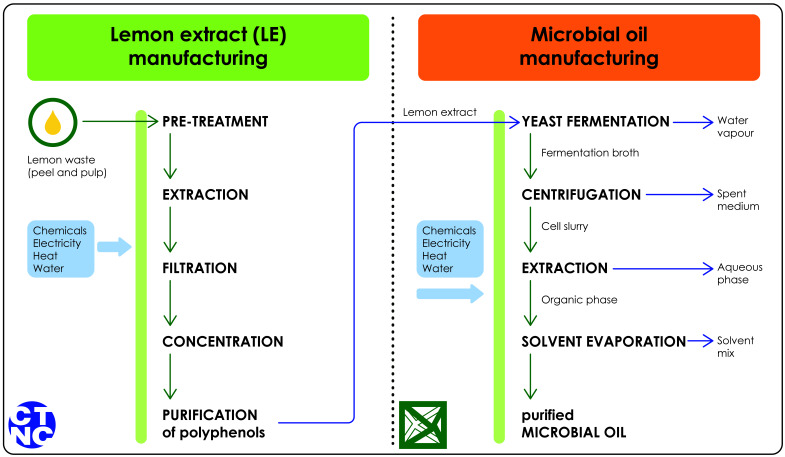
System boundary of the carbon footprint assessment. A detailed inventory table where the chemicals, electricity, heat and water consumptions are presented per process is available in the Supplementary material. Green arrows indicate the outputs; blue arrows indicate side streams.

The studied system is illustrated in Figure 1. This Figure describes the system at a high level, and more detailed LCI data is available in the supplementary materials ( **Sections 1 and 2**). Overall, the carbon footprint assessment is a cradle-to-gate study that consists of manufacturing the lemon extract and the microbial oil. The study excluded any infrastructure, transport and packaging. Infrastructure is typically excluded from LCA studies because its impact is generally insignificant ( European Commission, 2021). Transport and packaging were excluded because the study was an early-phase and partly laboratory-scale study.

***Scenarios***

Two scenarios were studied in the carbon footprint assessment. First, Spanish market electricity was used in all of the processes (scenario 1). In the second scenario (scenario 2) the electricity profile was switched to wind energy modeled with the ecoinvent 3.10 process electricity production, wind, 1–3 MW turbine, onshore, ES.

***Assumptions***

Assumptions were made to fill in the primary data gaps. The list of the used assumptions is available in the supplementary material ( **Section 2**).

***Allocation***

Allocation of the environmental burden between the processes was required in this study for lemon extract manufacturing. Mass-based allocation was used in the lemon enzymatic extraction where both liquid extracts (e.g. phenolic extract) and solid extracts (e.g. fiber extract) were produced. The intermediate product of the liquid phase was used to produce the microbial oil. The impacts from the mutual processes (pre-treatment, extraction, filtration) were calculated based on the share of produced liquid extract (0.95 kg phenolic extract and 3.6 kg fiber extract). The liquid phase produced was approximately 20%; hence, 20% of the impacts in the shared processes were allocated to the liquid phase. The possible environmental impacts from lemon production were excluded and allocated as zero for the used waste stream, lemon peel and pulp.

In the microbial oil manufacturing, allocation procedures were not applied and all the environmental burden was assigned to the microbial oil and its precursors due to the early stage and laboratory scale nature of the carbon footprint assessment.

***Limitations and uncertainties***

As this study was an early phase evaluation, it had various limitations. In the microbial oil manufacturing stage, laboratory-scale data from UNIMIB experiments and more upscaled data from CTNC were used together, which affected the interpretation of the results. The electricity consumption data was partly estimated from secondary data created based on the device models. Hence, the data in this life cycle stage contained various uncertainties.

***Data quality***

This study used primary data for lemon extract manufacturing processes. In microbial oil manufacturing, part of the electricity consumption data was based on secondary data of the typical electricity consumption of the used devices. Secondary data was also used to model the input chemicals, energy production and waste treatment emissions from ecoinvent database. Primary data from the solvent manufacturer was applied to model the solvents.

***Life cycle inventory***

The inventory tables are available in the supplementary material ( **Section 1**).

## Results and Discussion

### Characterization of sugar and nitrogen content of lemon extract

Lemon Extract (LE) was obtained from CTNC (Centro Tecnológico Nacional de la Conserva, Murcia, Spain) in sterile bags. Briefly, lemon waste (consisting mostly of peels and pulp) produced as residual biomass by the company Citromil (Murcia, Spain) was initially provided to CTNC for polyphenol extraction. The aqueous residues (LE) were processed to separate the solids by high-pressure filtration and stored in aseptic bags at 4 °C.

**Figure 2.**
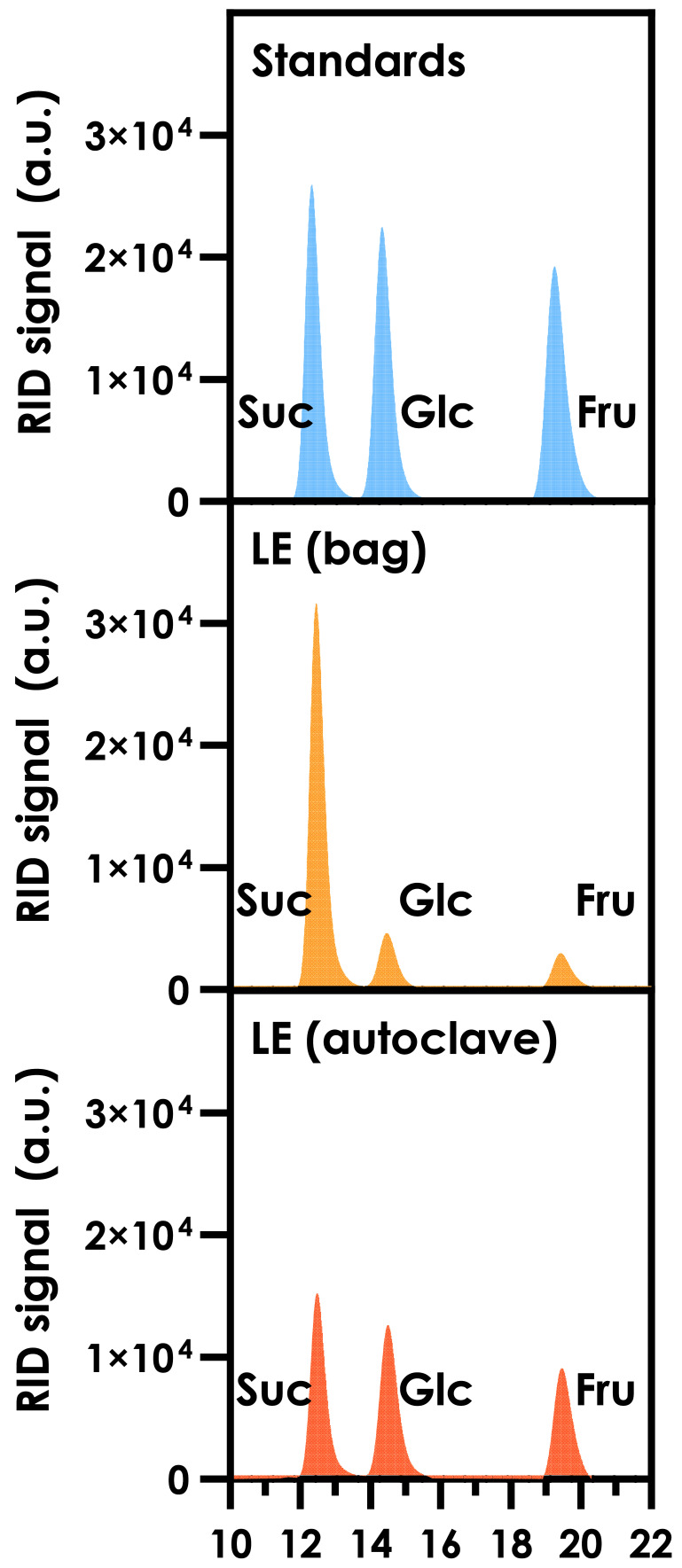
HPLC chromatograms obtained from the analysis of Lemon Extract (LE). The RID signal of the calibration standards (top panel) is shown in blue; the RID signals from the LE (bag) sample (middle) and LE (autoclave) sample (bottom) are shown in yellow and orange, respectively. Suc: sucrose; Glc: D-glucose; Fru: D-fructose.

Before the fermentation process, characterization of this side stream was necessary to identify and quantify the available fermentable sugars, as well as the nitrogen content. While the provided LE was already sterile and could theoretically be directly used for fermentation without any further processing, such an approach on a large scale would not be applicable. For this reason, we decided to assess the effect of autoclaving on LE to reproduce, at the lab scale, what would happen in a scaled-up process. For the identification and quantification of sugars, samples before and after the autoclavation step were analyzed by HPLC using a column for monosaccharides and sugars analysis. Three peaks were identified by comparison with known standards, corresponding to sucrose, glucose and fructose; no other peaks could be observed. The obtained chromatograms are shown in Figure 2; the full recorded chromatograms (from 0 to 30 min) are reported in **Figure S1**. To confirm the correct identification of the peaks, the samples were analyzed using a commercially available enzymatic kit for the quantification of glucose and fructose.

Quantification from both HPLC analysis and the enzymatic kits confirmed the presence of glucose and fructose in LE; most importantly, the quantification was consistent with both methods. The quantification obtained with the enzymatic kits is reported in Table 3.

**Table 3.** Composition of lemon extract (LE) before and after autoclaving. The pH, sugar content, nitrogen content and C/N ratio of LE were measured before (LE bag) and after (LE autoclave) the autoclaving step. See the main text for additional information. Glc: D-glucose; Fru: D-fructose.

	pH	Glc (g L ^-1^)	Fru (g L ^-1^)	YAN _tot_ (mg _N_ L ^-1^)	C/N
**LE (bag)**	3.90	10.49 ± 0.04	7.96 ± 0.24	290.14 ± 2.14	30
**LE (autoclave)**	3.99	28.77 ± 0.08	25.68 ± 0.27	298.44 ± 3.65	85

To confirm the sucrose peak, the samples were also analyzed by HPLC using a column for organic acids and fermentation by-products, and compared to calibration standards. Sucrose (as well as glucose and fructose) quantification was the same with both chromatographic methods (data not shown). A further confirmation of the correct identification of the sucrose peak was obtained by running the HPLC analysis at 80 °C: in these conditions, sucrose is partially hydrolyzed to glucose and fructose, due to the acidic mobile phase. Finally, analysis of samples with the organic acid HPLC column also revealed the presence of citric acid, which is to be expected when considering the source of LE.

Apart from the quantification of sugars, nitrogen content is also a key parameter to be considered when designing a fermentative process. In addition, lipid production is strictly related to the carbon to nitrogen (C/N) ratio of the medium. Indeed, lipid accumulation in oleaginous yeasts is triggered by nutrient limitation ( Abeln & Chuck, 2021) and nitrogen limitation is one of the most commonly exploited strategies to decouple cell growth and lipid accumulation. For these reasons, the total yeast available nitrogen (YAN _tot_) in LE was measured. Samples before and after the autoclaving step were analyzed with two commercially available enzymatic kits for the quantification of free ammonium, urea and L-arginine, and primary amino nitrogen (from amino acids). The obtained results ( Table 3) were utilized to calculate the C/N ratio of LE before and after the autoclavation step.

As it can be observed in Table 3, autoclavation of LE caused an increase in the concentrations of glucose and fructose and a decrease in the concentration of sucrose: the heat treatment in acidic conditions (pH < 4) caused the partial hydrolysis of sucrose. The same behavior can be observed in the HPLC chromatograms ( Figure 2), where the relative areas of the peaks are significantly different after the autoclavation step. On the other hand, no significant difference could be observed in terms of YAN _tot_, suggesting that the heat treatment does not affect the total yeast available nitrogen content. Thanks to the release of glucose and fructose, the autoclaved LE is characterized by an increased C/N ratio, compared to the LE from the bag. Indeed, sucrose was not considered in the calculations of the C/N ratio as no consumption of this sugar by *C. oleaginosum* was observed in preliminary tests (data not shown). Overall, these data suggest that autoclaving of LE is not detrimental; on the contrary, it increases the concentration of fermentable sugars and concomitantly the C/N ratio, which is beneficial for lipid accumulation. Thus, autoclaved LE was used for all the following growth experiments.

### Medium optimization for growth and lipid accumulation

After the characterization of the sugar and nitrogen content of LE, the substrate was assayed as a potential growth and production medium for *C. oleaginosum*. A simple medium optimization experiment was carried out. The objective was to identify (i) a condition to maximize growth and sugars consumption and (ii) a condition to maximize lipid accumulation. Indeed, the former is necessary for the expansion of pre-cultures, while the latter is required as the lipid production medium. Apart from the nitrogen content, growth might also be limited by the lack of other nutrients, such phosphates, sulphates, vitamins or trace metals. As described above, after autoclaving LE is characterized by a C/N ratio of 85: to balance the nitrogen content of the medium, urea was added as the nitrogen source to reach a C/N ratio of 8.8, which is the standard C/N ratio of synthetic minimal media used for growing yeasts ( Verduyn *et al.*, 1992); this medium is herein called LE8. Urea was chosen over ammonium sulfate as it has been shown that in batch fermentations the latter causes a significant drop in the pH during growth ( Mastella *et al.*, 2022). To account for the addition of the nutrients, autoclaved LE was slightly diluted (herein referred to as LE80). MgSO _4_ and KH _2_PO _4_ were added to both media (LE8.s and LE80.s) as a source of sulphates and phosphates, respectively. Finally, the addition of vitamins and trace elements was tested as well, either alone or in combination with the inorganic salts (LE8.v and LE80.v; LE8.vs and LE80.vs, respectively). Table 1 provides an overview of the tested media.

**Figure 3.**
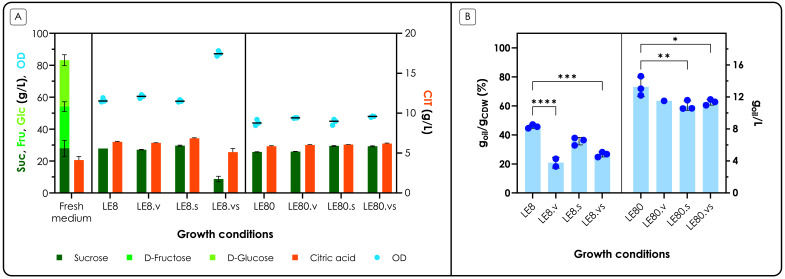
Growth and production medium optimization results. *C. oleaginosum* was cultivated in different LE media (see main text and Table 1 for more information), with the aim to maximize growth (LE8 media) or lipid accumulation (LE80 media). A. OD and carbon source concentrations in the medium at 72 h, compared to fresh medium. At the stop of the fermentation (72 h) the OD was measured at 600 nm; the supernatant was analyzed by HPLC to measure the concentration of sucrose (Suc), fructose (Fru), glucose (Glc) and citric acid (CIT). The x-axis shows the different growth conditions; the left y-axis shows the concentration of sucrose (dark green bars), fructose (green bars) and glucose (light green bars) in g L ^-1^ and OD (blue dots); bars are stacked. The right y-axis shows the concentration of citric acid (orange bars) in g L ^-1^. B. Lipid content at 72 h. At the end of the fermentation (72 h) total lipids were extracted from the pellets. The x-axis shows the different growth conditions; the left y-axis shows the percentage of oils of the accumulated biomass (% g _oil_ g _CDW_^-1^); the right y- axis shows the corresponding titer of oil per 1 mL of culture, expressed as a concentration (g _oil_ L ^-1^, blue dots). Values are the mean ± standard deviation of three independent experiments.

To assess the impact of the different media, *C. oleaginosum* was grown in 24-deepwell plates and growth was monitored over time. At the end of the lipid accumulation phase (72 h), the supernatant was analyzed to measure sugar and citric acid consumption; to assess lipid production, the cell pellets were extracted with the standard analytical Folch method ( Folch *et al.*, 1957) ( Figure 3).

At the end of the fermentation, glucose and fructose were found to be depleted in all conditions, both in LE8 and in LE80; interestingly, sucrose consumption was only observed in one condition, that is the balanced medium with inorganic salts, vitamins and trace elements (LE8.vs); coherently, the cells reached the highest OD obtained in the experiment (86 OD), compared to all other conditions. It is interesting to note that both inorganic salts, and vitamins and trace elements were required to allow the consumption of sucrose, as this behavior was not observed in either LE8.s or LE8.v. Most likely, nitrogen also plays a role, as in the corresponding limiting condition (LE80.vs) no sucrose consumption was observed. Citric acid consumption was not observed under any of these conditions. The growth profiles are shown in **Figure S2**. From these results, LE8.vs was selected as the medium for the pre-cultures, as it allowed the highest biomass accumulation and sugar consumption. The level of statistical significance is indicated only for differences between the samples.

In terms of lipid production, the cells grown in the balanced media (LE8-based media) accumulated a lower amount of lipids compared to the unbalanced media (LE80-based media), as expected. When considering the balanced conditions, addition of vitamins and trace elements (LE8.v, LE8.vs) significantly reduced lipid accumulation (P-values < 0.001). In nitrogen-limited conditions, however, it was the addition of salts (LE80.s, LE80.vs) that significantly reduced lipid accumulation (P-values < 0.05). Overall, the highest accumulation (0.73 ± 0.05 g _oil_ g _CDW_^-1^) was obtained with LE80, without the addition of any nutrient; anyhow, lipid accumulation was above 60% g _oil_ g _CDW_^-1^ in all other conditions with LE80. Most likely, LE80 allowed to reach the highest lipid content because some other nutrients (such as phosphates, vitamins or trace metals as observed with the supplemented LE80 media) became limiting, participating in increasing the cellular response to stress, and thus lipid accumulation ( Beopoulos *et al.*, 2011). For this reason, LE80 without any nutrient addition was selected as the production medium; this is also amenable from a process point of view, as no further additions are required for the fermentation, thus reducing the overall cost and preparation time.

### 2 L bioreactor fermentations for lipids production and extraction

After identifying the best production medium, the process was directly scaled-up to 2 L bioreactors for the production of lipids and the development of a suitable and simple downstream processing protocol. LE8.vs resulted in an effective pre-culture medium, allowing the accumulation of > 50 OD in less than 16 h of growth; moreover, upon inoculation, no lag phase was observed. The obtained fermentation profile is reported in **Figure S3, A**; after the exponential phase (12 h), the cells started accumulating lipids until the depletion of glucose and fructose, at 48 h; the two different growth phases can clearly be identified by the CO _2_ production and O _2_ consumption profiles ( **Figure S3, B**). At 48 h, 22.7 ± 2.2 g _CDW_ L ^-1^ of biomass were accumulated. After the depletion of the fermentable sugars, a drop in the cell dry weight was observed (19.6 ± 0.3 g _CDW_ L ^-1^), possibly due to the cells starting the consumption of the accumulated lipid. The fermentation was stopped (52 h) and the broth from the two reactors was collected for lipid extraction. Overall, the cells accumulated 0.47 ± 0.08 g _oil_ g _CDW_^-1^, in a relatively short process time. This result is significantly lower than the accumulation obtained in the medium optimization experiment. One possible explanation, in addition to the one mentioned above, can be given by the controlled environment in the bioreactor: maintenance of optimal pH and oxygenation might be reducing the stress conditions, and thus the overall lipid accumulation.

Lipid extraction is usually the most complex and costly part of microbial oil production, and it often requires toxic chemicals ( Ugur *et al.*, 2024). Indeed, the literature standard methods (Folch method, Bligh and Dyer) are based on a mixture of MeOH/CHCl _3_ ( Bligh & Dyer, 1959; Bonturi *et al.*, 2015; Folch *et al.*, 1957). MeOH and CHCl _3_ are both highly toxic and harmful: MeOH is known to cause damage to organs (H370) with practically every know exposure route, while CHCl _3_ is also suspected of causing cancer (H351), just to name a couple of recognized chemical risks to work with these substances (GHS06 and GHS08). These solvents should be avoided for commercial use, as this would hinder the use of such lipids in the sectors where they could benefit the most: cosmetic and pharma. The most common alternative to ensure lipid extraction is the use of a combination of polar and non-polar solvents ( Breil *et al.*, 2017; Ugur *et al.*, 2024). Another variable in the process is the drying of the yeast biomass: usually dry biomass improves the extraction yield, but comes at a greater cost in terms of energy consumption and time.

To find an alternative extraction method tailored to our specific process, we compared the standard Folch method with five different pairs of solvents blends provided by the ASTROBIO™ Green Solvents Division of Soft Chemicals S.r.l. ASTROBIO™ BA was employed as an alternative to MeOH; the solvents ASTROBIO™ XT, SD, K1 and K100 (more apolar), on the other hand, were proposed as an alternative to CHCl _3_. Finally, ethanol and ethyl acetate were also tested as potential candidates, as this pair was selected by Breil and colleagues in an in depth study aimed at lipid extraction optimization from *Yarrowia lipolytica* ( Breil *et al.*, 2017). All ASTROBIO™ Green Solvents tested, as well as ethanol and ethyl acetate, are just recognized to be irritant (GHS07), thus making them a more suitable option for large-scale applications.

All solvents were tested with both wet and dry samples to assess whether the additional drying step would increase the extraction yield (as in the traditional protocol). The test was performed on a small scale using a sample of the fermentation broth (see Materials and Methods section for more details). Table 4 summarizes the results. As expected, the Folch method performed best with both dried and wet samples; the obtained lipid yield showed no significant difference between the two strategies. The best green solvent alternative resulted in the pair BA/K1, which showed an extraction efficacy of 78% (for dry samples) and 79% (for wet samples) when compared to the MeOH/CHCl _3_ (Folch) method. BA/XT and BA/K100 were also effective but showed slightly lower efficiency with respect to BA/K1, especially when extracting from wet samples. Interestingly, the optimized EtOH/EtOAc pair performed worse than any of the tested pairs from ASTROBIO™, except for BA/SD, which showed the lowest efficiency with dried samples (16%) and a large variability (approximately 30%) for wet samples. Based on these considerations, BA/K1 was selected as the solvent for lipid extraction at a larger scale. As no significant differences were observed between dry and wet samples, the wet extraction method was selected.

**Table 4.** Small scale extraction trials with green solvents. The table shows the obtained lipid content (% g _oil_ g _CDW_^-1^) with each extraction method and its efficiency compared to the MeOH/CHCl _3_ (Folch) method. MeOH: methanol; CHCl _3_: chloroform; EtOH: ethanol; EtOAc: ethyl acetate. Values are the mean ± standard error of two independent experiments.

	Solvent pair	Lipid content (% g _oil_/g _CDW_)	Efficiency compared to MeOH/CHCl _3_
**dried samples**	MeOH/CHCl _3_	47.03% ± 7.9%	100%
	EtOH/EtOAc	29.4% ± 4.51%	62.67% ± 0.93%
	BA/XT	35.37% ± 0.31%	77.5% ± 13.69%
	BA/SD	7.37% ± 0.58%	15.91% ± 1.43%
	**BA/K1**	**36.67% ± 6.27%**	**77.92% ± 0.24%**
	BA/K100	35.6% ± 1.54%	77.33% ± 9.72%
**wet samples**	MeOH/CHCl _3_	52.3% ± 6.83%	100%
	EtOH/EtOAc	28.17% ± 2.56%	54.14% ± 2.17%
	BA/XT	33.41% ± 4.27%	63.9% ± 0.18%
	BA/SD	56.85% ± 23.73%	104.55% ± 31.73%
	**BA/K1**	**41.01% ± 3.04%**	**79.00% ± 4.49%**
	BA/K100	30.14% ± 2.32%	58.03% ± 3.13%

**Figure 4.**
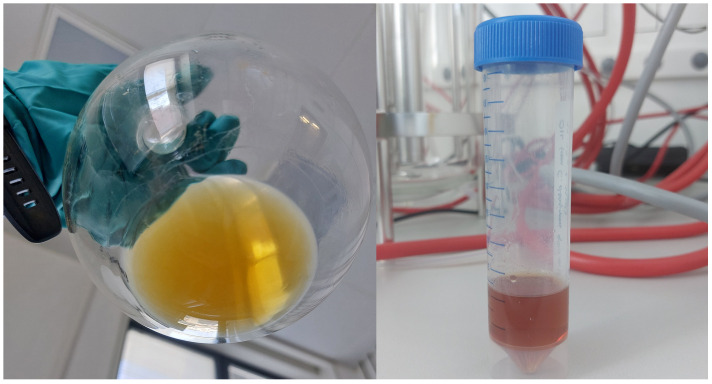
Extracted oil from *C. oleaginosum* grown on LE.

For large-scale extraction, our goal was to design a simple downstream process. First, the biomass was pre-treated by freezing at -20 °C (a common temperature for storage) and thawing before extraction, in an effort to increase the accessibility of the solvents to the lipid structure ( Meullemiestre *et al.*, 2016). The biomass obtained from the two bioreactors was combined, and the extraction was carried out in a beaker. Overall, 11.29 g of oil were obtained, corresponding to 35% of the dry weight of the biomass. The extraction yield is well in line with the results obtained in the 1 mL scale (0.41 ± 0.03 g _oil_ g _CDW_^-1^), indicating that our extraction protocol is effective and robust to the scale-up. Figure 4 shows a picture of the obtained oil.

### Carbon footprint - impact assessment

**Figure 5.**
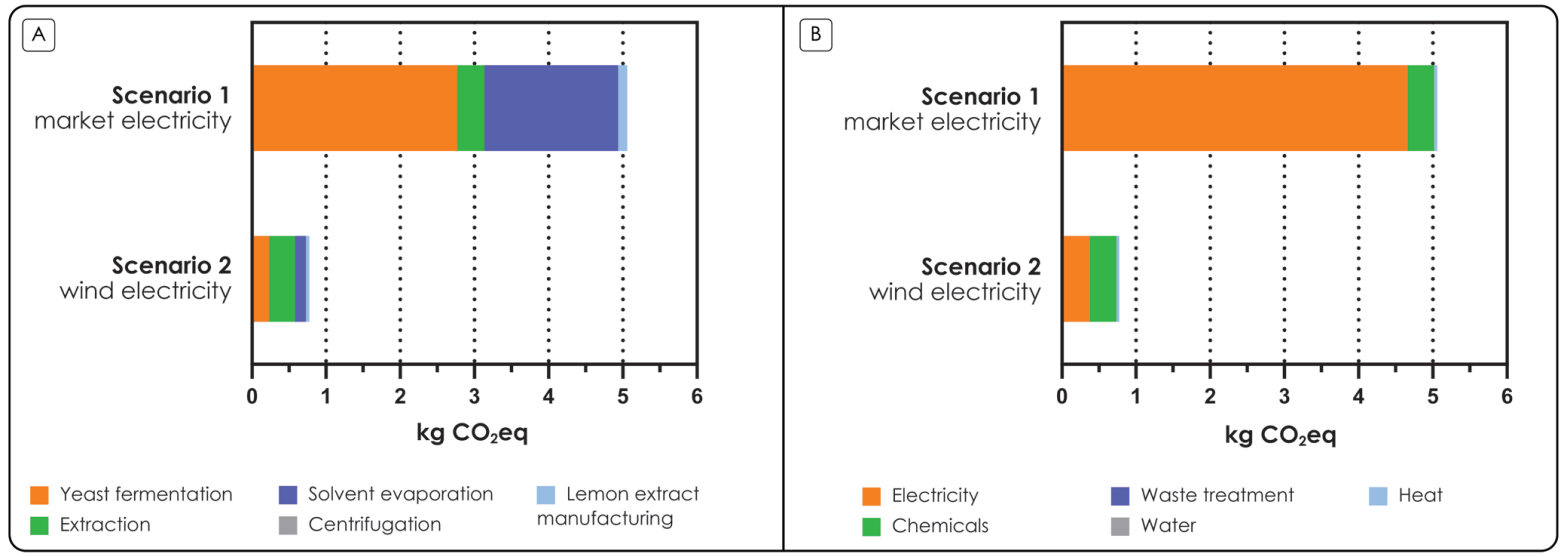
Estimated carbon footprints. **A**. Estimated carbon footprint of 11.79 g of the laboratory-scale microbial oil from lemon extract per life cycle stage with market (Scenario 1) and wind (Scenario 2) electricity in all the processes. **B**. Estimated carbon footprint of 11.79 g of the laboratory-scale microbial oil from lemon extract per process type with market (Scenario 1) and wind (Scenario 2) electricity in all the processes.

As shown in Figure 5, A in scenario 1, the estimated carbon footprint of producing 11.79 g of microbial oil from lemon extract was 5.12 kgCO _2_eq if Spanish market electricity was applied. From the same figure, it can be seen that the life cycle stages “Yeast fermentation” and “Solvent evaporation” contributed the most to the carbon footprint with shares of 54% and 35%, respectively. This was due to the intense electricity consumption in these two stages. The lemon extract manufacturing did not significantly impact the carbon footprint, covering only 2% of the total carbon footprint in Scenario 1. The dominance of the emissions from electricity consumption is also visible in Figure 5, B where electricity consumption comprised 91% of the total carbon footprint in Scenario 1.

In scenario 2, where we hypothesized that wind electricity was used, the carbon footprint of producing 11.79 g of microbial oil from lemon extract was 0.837 kgCO _2_eq ( Figure 5, A). In this scenario, the life cycle stage “Extraction” contributed the most (50%) to the total carbon footprint of scenario 2, due to the consumption of the solvents. On process type level, Figure 5, B demonstrates that consumption of electricity and chemicals contributed most to the carbon footprint, 44% and 51% of the total carbon footprint, respectively. In this scenario, the electricity emissions are the upstream emissions of the wind electricity, and the most contributing chemicals are the solvents, similarly to Scenario 1.

In this study, the carbon footprint of lemon extract was 71 – 434 kgCO _2_eq kg ^-1^ microbial oil, depending on the electricity profile. In previous yeast-derived microbial oil studies the carbon footprints have ranged from 3.56 kgCO _2_eq kg ^-1^ of yeast oil ( Masri *et al.*, 2019) to 26.94 kgCO _2_eq kg ^-1^ of microbial oil ( Abelleira *et al.*, 2024). Previous LCA studies differ from the carbon footprint calculation presented in this article in terms of technology maturity level, utilization of co-products, and circulating materials. Masri *et al.* (2019) performed a cradle-to-gate LCA of a commercial-scale yeast lipid production unit. In this study, all side-product streams were recycled within the processes. Allocation procedures were not applied. Abelleira *et al.* (2024) carried out a cradle-to-gate LCA where the fermenters had volumes ranging from 74 to 354 m ^3^. The process system contained loops where the side streams were reapplied within the system. Furthermore, 89.4% of the system burden, which also had a waste management function, was allocated for the microbial oil. In this study, the input organic waste feedstock was assumed to be burden-free.

The environmental impact of microbial oil was also compared to the impact of conventional vegetable oils. More specifically, coconut oil was chosen as the conventional benchmark product because the microbial oil produced in the A2C project has been applied in the cosmetic industry. In the ecoinvent database the carbon footprint of crude coconut oil is 2.58 kgCO _2_eq. Crude coconut oil was used as a comparison because the microbial oil manufactured in this study also requires further processing before it can be applied in a cosmetic product.

The calculated carbon footprint in this study was higher compared to the carbon footprint values in other studies and in the database data of a conventional vegetable oil. This is expected to be explained by the laboratory scale of the experiment and the uncertainties related to the electricity consumption.

The carbon footprint study was an early-stage estimation based on combining pilot-scale data of lemon extract manufacturing with laboratory-level data of microbial oil manufacturing. The consumption of electricity and solvents in the microbial oil manufacturing stage dominated the results. However, the electricity consumption values in the microbial oil manufacturing were partially estimated. If market electricity is applied, the carbon footprint of the microbial oil manufacturing should decrease 168 times to reach the carbon footprint of the crude coconut oil. To do this, the electricity consumption and the chemical requirements would need to be significantly reduced in the scaling-up processes.

The following section presents calculations on the potential decrease in electricity consumption if the fermenter scale increases from 2 L to 300 L. Full calculations are available in Supplementary material section 4. In the A2C trials, 300 L reactors were used for a fermentation of similar duration to our lab scale process (48 h). Assuming that yield and productivity remain the same for microbial oil production, a 300 L fermenter would require 2.13 kWh/kg microbial oil, whereas the 2 L fermenter required 346.9 kWh/kg microbial oil. Hence, the electricity consumption of the 300 L fermenter is 163 times smaller than the 2 L fermenter.

However, the power consumption of the fermentation step is only one aspect of the environmental impact of the process. Indeed, even if the share of electricity consumption emissions were minimized, as in scenario 2 ( Figure 5, B) or with scale-up, there would still be an environmental burden arising from the manufacturing of solvents and chemicals. The carbon footprint of the chemicals and solvents was 36.0 kgCO _2_eq/kg microbial oil. This is significantly higher than all the benchmarks listed by Masri *et al.* (2019) and Abelleira *et al.* (2024) and more than 14 times higher than the carbon footprint of coconut oil. Therefore, it is essential to reduce chemical and solvent emissions through efficient recycling of chemicals and, most importantly, by improving the yield through precise monitoring of the fermentation parameters. Indeed, regarding the last aspect, we showed that it is theoretically possible for *C. oleaginosum* to accumulate at least up to 0.73 ± 0.05 g _oil_ g _CDW_^-1^, which is 50% more product compared to the 2 L fermentation.

To evaluate the carbon footprint of the process in a more robust manner, a scale-up to the industrial level of the microbial oil production system and downstream procedures is required to gather precise information on production processes, electricity consumption, and chemical emissions.

## Conclusions

The aim of this work was to develop a fermentation process for the upcycling of an aqueous LE side stream rich in sugars derived from the extraction of polyphenols from waste lemon peel and pulp into microbial oil.

The first part of this work focused on the characterization of LE from the point of view of the sugars and nitrogen compositions, two fundamental parameters for yeast growth and lipid accumulation. Autoclaving of LE resulted in hydrolysis of sucrose, and therefore with an advantageous increase in the glucose and fructose concentrations. After the initial characterization, LE composition was optimized to maximize either growth or lipid accumulation of *C. oleaginosum*. A screening of different growth conditions identified LE8.vs as the ideal medium for growth, while LE80 for production. Interestingly, sucrose was only consumed in one of the tested conditions. The highest lipid production obtained was 0.74 ± 0.05 g _oil_ g _CDW_^-1^. Overall, it was found that autoclaved LE is with minor adjustments the optimal medium for sustaining yeast oil production.

From the promising results obtained on the small scale, *C. oleaginosum* was cultivated in 2 L bioreactors and the obtained biomass was utilized for the development of a sustainable and easily scalable downstream protocol for the extraction of lipids, by comparing different extraction protocols. Our findings confirmed - as expected - that the traditionally used Folch method is the best at extracting lipids; however, three pairs of green solvents showed promising results as alternatives to methanol and chloroform, the best one being ASTROBIO™ BA/K1 (Soft Chemicals S.r.l.) with an efficiency of almost 80% when compared to the Folch method. Scale-up of the extraction process from 1.5 mL tubes to the biomass collected from the bioreactors showed consistent results and allowed to recover 11.29 g of oil, corresponding to 35% of the cell dry weight. When considering the overall process, the oil yield from sugars was 0.14 g _oil_ g _S_^-1^; this result is promising, but further optimizations are required for an economically viable process.

Future studies should focus on the improvement of fermentation parameters (pH, oxygenation, temperature), to increase the lipid content of the cells, similarly to what was obtained on the small-scale during media optimization. Additionally, the complete hydrolysis of sucrose (or further media optimization) could increase the total amount of fermentable sugars, and consequently the theoretical maximum oil yield.

In this study, the carbon footprint of microbial oil from lemon extract was 71 – 434 kgCO _2_eq/kg microbial oil depending on the electricity profile. The consumption of electricity and solvents in the microbial oil manufacturing stage dominated the results. The carbon footprint study was an early-stage estimation based on combining pilot-scale data of lemon extract manufacturing with laboratory-level data of microbial oil manufacturing. The calculations should be updated when additional scaled-up data becomes available. It is also relevant to note that, with respect to other examples of oil production, this process valorizes a secondary residual biomass, which does not require complex, costly or time-consuming treatment to be used as feedstock. Therefore, it is well in line with the strategic action plan of the EU entitled *“Building the future with nature: Boosting Biotechnology and Biomanufacturing in the EU”*, providing a feasible example of maximizing the use of planetary resources, both in terms of value and time.

A preliminary estimation of the hypothetical carbon footprint reduction of the microbial oil manufacturing was performed using scaled-up data on electricity consumption. The estimation showed that the electricity consumption of a larger fermenter is significantly reduced, but also that improvements in the solvent and chemical consumption, and production yield must take place to potentially reach the order of the carbon footprint of currently existing solutions. However, as the estimation was based on the electricity consumption of the fermenter only instead of the full equipment, the results should only be considered indicative. As more robust data on the complete microbial oil production is available, it is suggested to perform a comprehensive LCA study where various impact categories are included to detect possible trade-offs between life cycle stages and impact categories.

## Ethics and consent

Ethical approval and consent were not required.

## Data Availability

Bicocca Open Archive Research Data: Production and carbon footprint of microbial oil from waste lemon peel extract - supplementary material, https://doi.org/10.17632/bsc44zsfdd.2 [ Senatore & Branduardi, 2025]. *This project contains the following underlying data:* *[Supplementary data.pdf] (The provided file contains the Supplementary Data, consisting of Life Cycle Inventory, LCI assumptions, Figure S1, Figure S2, Figure S3 and the calculations for microbial oil manufacturing electricity consumption upscaling).* *[Underlying data.xlsx] (The provided file includes the values behind the means, standard deviations and other measures reported, as well as all the other values used to build all the graphs and all the tables in the manuscript and in the Supplementary data).* Data are available under the terms of the Creative Commons Attribution 4.0 International license (CC-BY 4.0).

## References

[ref-1] Abelleira S Cruz PL Iribarren D : Life cycle sustainability assessment of microbial oil from organic waste. *Clean Environ Syst.* 2024;15: 100236. 10.1016/j.cesys.2024.100236

[ref-2] Abeln F Chuck CJ : The history, state of the art and future prospects for oleaginous yeast research. *Microb Cell Fact.* 2021;20(1): 221. 10.1186/s12934-021-01712-1 34876155 PMC8650507

[ref-3] Balina K Soloha R Suleiko A : Prospective life cycle assessment of microbial sophorolipid fermentation. *Fermentation.* 2023;9(9):839. 10.3390/fermentation9090839

[ref-4] Beopoulos A Nicaud JM Gaillardin C : An overview of lipid metabolism in yeasts and its impact on biotechnological processes. *Appl Microbiol Biotechnol.* 2011;90(4):1193–1206. 10.1007/s00253-011-3212-8 21452033

[ref-5] Bligh EG Dyer WJ : A rapid method of total lipid extraction and purification. *Can J Biochem Physiol.* 1959;37(8):911–917. 10.1139/o59-099 13671378

[ref-6] Bonatsos N Marazioti C Moutousidi E : Techno-economic analysis and life cycle assessment of heterotrophic yeast-derived single cell oil production process. *Fuel.* 2020;264: 116839. 10.1016/j.fuel.2019.116839

[ref-7] Bonturi N Matsakas L Nilsson R : Single cell oil producing yeasts *Lipomyces starkeyi* and *Rhodosporidium toruloides*: selection of extraction strategies and biodiesel property prediction. *Energies.* 2015;8(6):5040–5052. 10.3390/en8065040

[ref-8] Branduardi P : Closing the loop: the power of microbial biotransformations from traditional bioprocesses to biorefineries, and beyond. *Microb Biotechnol.* 2021;14(1):68–73. 10.1111/1751-7915.13713 33275324 PMC7888447

[ref-9] Breil C Abert Vian M Zemb T : "Bligh and Dyer" and Folch methods for solid–liquid–liquid extraction of lipids from microorganisms. Comprehension of solvatation mechanisms and towards substitution with alternative solvents. *Int J Mol Sci.* 2017;18(4):708. 10.3390/ijms18040708 28346372 PMC5412294

[ref-10] Chopra J Tiwari BR Dubey BK : Environmental impact analysis of oleaginous yeast based biodiesel and bio-crude production by life cycle assessment. *J Clean Prod.* 2020;271: 122349. 10.1016/j.jclepro.2020.122349

[ref-11] Di Fidio N Minonne F Antonetti C : *Cutaneotrichosporon oleaginosum*: a versatile whole-cell biocatalyst for the production of single-cell oil from agro-industrial wastes. *Catalysts.* 2021;11(11):1291. 10.3390/catal11111291

[ref-12] European Commission: COMMISSION RECOMMENDATION of 16.12.2021 on the use of the Environmental Footprint methods to measure and communicate the life cycle environmental performance of products and organisations. Brussels: Directorate-General for Environment,2021. Reference Source

[ref-13] Folch J Lees M Sloane Stanley GH : A simple method for the isolation and purification of total lipides from animal tissues. *J Biol Chem.* 1957;226(1):497–509. 10.1016/S0021-9258(18)64849-5 13428781

[ref-14] Gontard N Sonesson U Birkved M : A research challenge vision regarding management of agricultural waste in a circular bio-based economy. *Crit Rev Environ Sci Technol.* 2018;48(6):614–654. 10.1080/10643389.2018.1471957

[ref-16] Kourist R Bracharz F Lorenzen J : Genomics and transcriptomics analyses of the oil-accumulating basidiomycete yeast *Trichosporon oleaginosum*: insights into substrate utilization and alternative evolutionary trajectories of fungal mating systems. *mBio.* 2015;6(4): e00918. 10.1128/mBio.00918-15 26199329 PMC4513080

[ref-17] Longati AA Campani G Furlan FF : Microbial oil and biodiesel production in an integrated sugarcane biorefinery: techno-economic and life cycle assessment. *J Clean Prod.* 2022;379(Part 2): 134487. 10.1016/j.jclepro.2022.134487

[ref-18] Martínez-Abad A Ramos M Hamzaoui M : Optimisation of sequential microwave-assisted extraction of essential oil and pigment from lemon peels waste. *Foods.* 2020;9(10):1493. 10.3390/foods9101493 33086617 PMC7603390

[ref-19] Masri MA Garbe D Mehlmer N : A sustainable, high-performance process for the economic production of waste-free microbial oils that can replace plant-based equivalents. *Energy Environ Sci.* 2019;12(9):2717–2732. 10.1039/c9ee00210c

[ref-20] Mastella L Senatore VG Guzzetti L : First report on Vitamin B _9_ production including quantitative analysis of its vitamers in the yeast *Scheffersomyces stipitis*. *Biotechnol Biofuels Bioprod.* 2022;15(1): 98. 10.1186/s13068-022-02194-y 36123695 PMC9487109

[ref-40] Meullemiestre A Breil C Abert-Vian M : Microwave, ultrasound, thermal treatments, and bead milling as intensification techniques for extraction of lipids from oleaginous Yarrowia lipolytica yeast for a biojetfuel application. *Bioresour Technol.* 2016;211:190–9. 10.1016/j.biortech.2016.03.040 27017129

[ref-21] Ogbu CC Okey SN : Agro-industrial waste management: the circular and bioeconomic perspective. In: *Agricultural Waste—New Insights*. IntechOpen,2023. 10.5772/intechopen.109181

[ref-22] Sae-ngae S Cheirsilp B Louhasakul Y : Techno-economic analysis and environmental impact of biovalorization of agro-industrial wastes for biodiesel feedstocks by oleaginous yeasts. *Sustain Environ Res.* 2020;30(1): 11. 10.1186/s42834-020-00052-w

[ref-24] Senatore VG Branduardi P : Production and carbon footprint of microbial oil from waste lemon peel extract - supplementary material. Bicocca Open Archive Research Data, V1. 2025. 10.17632/bsc44zsfdd.1 PMC12856252 41624330

[ref-23] Senatore VG Milanesi R Masotti F : Exploring yeast biodiversity and process conditions for optimizing ethylene glycol conversion into glycolic acid. *FEMS Yeast Res.* 2024;24: foae024. 10.1093/femsyr/foae024 39104224 PMC11344169

[ref-25] Sharma T Dasgupta D Singh J : Yeast lipid-based biofuels and oleochemicals from lignocellulosic biomass: life cycle impact assessment. *Sustain Energy Fuels.* 2020;4(1):387–398. 10.1039/c9se00540d

[ref-26] Sulca: Sustainability tool for ecodesign, footprint calculations & LCA.2024. Reference Source

[ref-27] Uğur Ş Zieniuk B Fabiszewska A : Nutritional and medicinal properties of microbial oil. *Appl Sci.* 2024;14(10):4232. 10.3390/app14104232

[ref-28] Vera L Sun W Iftikhar M : LCA based comparative study of a microbial oil production starch wastewater treatment plant and its improvements with the combination of CHP system in Shandong, China. *Resour Conserv Recycl.* 2015;96:1–10. 10.1016/j.resconrec.2014.09.013

[ref-29] Verduyn C Postma E Scheffers WA : Effect of benzoic acid on metabolic fluxes in yeasts: a continuous-culture study on the regulation of respiration and alcoholic fermentation. *Yeast.* 1992;8(7):501–517. 10.1002/yea.320080703 1523884

[ref-30] Zhu Y Luan Y Zhao Y : Current technologies and uses for fruit and vegetable wastes in a sustainable system: a review. *Foods.* 2023;12(10):1949. 10.3390/foods12101949 37238767 PMC10217424

