# Supplementary material for: Production and carbon footprint of microbial oil from waste lemon peel extract
Source: Open Res Eur. 2026 Jan 22;5:261. Originally published 2025 Aug 28. [Version 2] doi: 10.12688/openreseurope.20856.2 (PMC12856252; doi:10.12688/openreseurope.20856.2)
Supplement: Peer review history

# Reviewer report: Reviewer response for version 2

**DOI:** 10.21956/openreseurope.23890.r68552

**Published:** 2026-01-30

**Authors:** Luis Alfonso Díaz-Secades

**Affiliations:** 1 Universidad de Oviedo, Oviedo, Spain

I approve this new version as it is satisfactory, I have no further comment.

Is the study design appropriate and does the work have academic merit?

Yes

Is the work clearly and accurately presented and does it cite the current literature?

Partly

If applicable, is the statistical analysis and its interpretation appropriate?

Partly

Are all the source data underlying the results available to ensure full reproducibility?

Yes

Are the conclusions drawn adequately supported by the results?

Partly

Are sufficient details of methods and analysis provided to allow replication by others?

Partly

Reviewer Expertise:

NA

I confirm that I have read this submission and believe that I have an appropriate level of expertise to confirm that it is of an acceptable scientific standard.

# Reviewer report: Reviewer response for version 2

**DOI:** 10.21956/openreseurope.23890.r68553

**Published:** 2026-01-29

**Authors:** Wilailuk Niyommaneerat

**Affiliations:** 1 Sustainable Environment Research Institute, Chulalongkorn University, Bangkok, Bangkok, Thailand

. I approved for the revision and no further comment.

Is the study design appropriate and does the work have academic merit?

No

Is the work clearly and accurately presented and does it cite the current literature?

Yes

If applicable, is the statistical analysis and its interpretation appropriate?

I cannot comment. A qualified statistician is required.

Are all the source data underlying the results available to ensure full reproducibility?

Partly

Are the conclusions drawn adequately supported by the results?

Yes

Are sufficient details of methods and analysis provided to allow replication by others?

Yes

Reviewer Expertise:

NA

I confirm that I have read this submission and believe that I have an appropriate level of expertise to confirm that it is of an acceptable scientific standard.

# Reviewer report: Reviewer response for version 1

**DOI:** 10.21956/openreseurope.22564.r60226

**Published:** 2025-09-30

**Authors:** Rarosue J Amaraibi

**Affiliations:** 1 National Renewable Energy Laboratory (Ringgold ID: 53405), Golden, Colorado, USA; 2 University of South Florida, Tampa, Florida, USA

**SUMMARY**

 This manuscript presents a fermentation process for converting lemon peel extract (LE), a waste stream from polyphenol extraction, into microbial oil using the oleaginous yeast *Cutaneotrichosporon oleaginosum*. The study includes three main components: (1) characterization and optimization of LE as a fermentation medium, (2) development of a green solvent extraction method, and (3) carbon footprint assessment of the process.

 Key findings include autoclaving LE to hydrolyze sucrose into fermentable glucose and fructose, achieving lipid accumulation of 0.47 ± 0.08 g_oil/g_CDW in 2L bioreactors, and developing an extraction method using ASTROBIO™ BA/K1 solvents with 79% efficiency compared to the traditional Folch method. The carbon footprint assessment reported 71-434 kgCO₂eq per kg microbial oil (depending on electricity source), significantly higher than conventional coconut oil at 2.58 kgCO₂eq/kg.

**CRITICAL ISSUES REQUIRING CORRECTION**

**1. Life Cycle Assessment Functional Unit**

**Issue:** The functional unit is stated as "11.79 g of microbial oil," which violates LCA standardization conventions and makes results non-comparable with existing literature.

**Required correction:** The functional unit must be restated as **1 kg of microbial oil** (or alternatively 1 g, though 1 kg is standard practice). This is a fundamental requirement for scientifically valid LCA reporting per ISO 14040/14044 standards and enables direct comparison with published microbial oil and vegetable oil studies.

**2. Scale-Up Modeling and Data Quality**

**Issue:** The study combines laboratory-scale (2L) primary data with speculative industrial-scale (300L) calculations without rigorous process modeling. The electricity consumption reduction claim (163-fold) is based solely on fermenter volume scaling without accounting for downstream processing, utilities, and auxiliary equipment.

**Required corrections:**

- Either conduct comprehensive process simulation using software (Aspen Plus, SuperPro Designer) for industrial-scale material and energy balances, OR
- Clearly restrict the scope to "laboratory-scale environmental screening" and remove direct comparisons with industrial vegetable oil production
- Add explicit uncertainty ranges for all scaled-up estimates
- Include all unit operations (not just fermentation) in scale-up calculations
- If direct comparison with industrial coconut oil (2.58 kgCO₂eq/kg) is retained, explicitly state: "This laboratory-scale carbon footprint is 27-168 times higher than industrial vegetable oil production, indicating substantial optimization is required before environmental competitiveness can be achieved"

**3. Incomplete Sensitivity Analysis**

**Issue:** Only electricity source variation is analyzed. Critical parameters that substantially affect environmental impact are omitted.

**Required additions:**

- Lipid extraction efficiency (current 35% vs. theoretical 73% achieved in optimization experiments)
- Fermentation yield variations (0.47 vs. 0.73 g_oil/g_CDW)
- Solvent recovery and recycling rates (currently assumed zero recovery, yet solvents contribute 36.0 kgCO₂eq/kg or 51% of scenario 2 impact)
- C/N ratio optimization effects on yield
- Plant capacity scenarios (batch size effects)

Present results showing relative impact of each parameter on the overall carbon footprint to identify which factors most critically affect environmental performance and where optimization efforts should focus.

**RECOMMENDATION**

 Major revisions required to address the three issues above. The fermentation and extraction optimization work is solid, but the LCA component requires substantial methodological corrections to meet scientific standards.

Is the study design appropriate and does the work have academic merit?

Yes

Is the work clearly and accurately presented and does it cite the current literature?

Yes

If applicable, is the statistical analysis and its interpretation appropriate?

Partly

Are all the source data underlying the results available to ensure full reproducibility?

Partly

Are the conclusions drawn adequately supported by the results?

Partly

Are sufficient details of methods and analysis provided to allow replication by others?

Yes

Reviewer Expertise:

Process modeling and simulation, techno-economic analysis, life cycle assessment (LCA) and carbon footprint analysis, industrial scale-up of sustainable technologies, waste valorization, and circular economy systems.

I confirm that I have read this submission and believe that I have an appropriate level of expertise to confirm that it is of an acceptable scientific standard, however I have significant reservations, as outlined above.

# Response

**Published:** 2025-11-27

**Authors:** Vittorio Giorgio Senatore

**Comment: **This manuscript presents a fermentation process for converting lemon peel extract (LE), a waste stream from polyphenol extraction, into microbial oil using the oleaginous yeast *Cutaneotrichosporon oleaginosum.* The study includes three main components: (1) characterization and optimization of LE as a fermentation medium, (2) development of a green solvent extraction method, and (3) carbon footprint assessment of the process. Key findings include autoclaving LE to hydrolyze sucrose into fermentable glucose and fructose, achieving lipid accumulation of 0.47 ± 0.08 g_oil/g_CDW in 2L bioreactors, and developing an extraction method using ASTROBIO™ BA/K1 solvents with 79% efficiency compared to the traditional Folch method. The carbon footprint assessment reported 71-434 kgCO₂eq per kg microbial oil (depending on electricity source), significantly higher than conventional coconut oil at 2.58 kgCO₂eq/kg.   CRITICAL ISSUES REQUIRING CORRECTION 1. Life Cycle Assessment Functional Unit Issue: The functional unit is stated as "11.79 g of microbial oil," which violates LCA standardization conventions and makes results non-comparable with existing literature. Required correction: The functional unit must be restated as 1 kg of microbial oil (or alternatively 1 g, though 1 kg is standard practice). This is a fundamental requirement for scientifically valid LCA reporting per ISO 14040/14044 standards and enables direct comparison with published microbial oil and vegetable oil studies.  

**Response: **We thank the reviewer for their very useful suggestion. The functional unit was changed to 1 kg (Page 8). All the corrections to the values are marked with red font throughout the carbon footprint calculation.   

**Comment: **2. Scale-Up Modeling and Data Quality Issue: The study combines laboratory-scale (2L) primary data with speculative industrial-scale (300L) calculations without rigorous process modeling. The electricity consumption reduction claim (163-fold) is based solely on fermenter volume scaling without accounting for downstream processing, utilities, and auxiliary equipment.   Required corrections:

- Either conduct comprehensive process simulation using software (Aspen Plus, SuperPro Designer) for industrial-scale material and energy balances, OR
- Clearly restrict the scope to "laboratory-scale environmental screening" and remove direct comparisons with industrial vegetable oil production
- Add explicit uncertainty ranges for all scaled-up estimates
- Include all unit operations (not just fermentation) in scale-up calculations
- If direct comparison with industrial coconut oil (2.58 kgCO₂eq/kg) is retained, explicitly state: "This laboratory-scale carbon footprint is 27-168 times higher than industrial vegetable oil production, indicating substantial optimization is required before environmental competitiveness can be achieved"

**Response: **We thank the reviewer for the comments and suggestions. Regarding the scale-up calculations, we do agree that the data is too speculative. For this reason, we changed that part to be more conservative:   Page 16 If market electricity is applied, the carbon footprint of the microbial oil manufacturing should decrease 168 times to reach the carbon footprint of the crude coconut oil. To do this, the electricity consumption and the chemical requirements would need to be significantly reduced in the scaling-up processes. While the required decrease is substantial, it is not unrealistic: large scale fermenters require far less power per unit of volume if compared to benchtop bioreactors. As an example, the electricity consumption (per liter) of a 300 L fermenter of one of the partners of the A2C project is 160 times smaller than the one required by the 2L bioreactors used in study. From these observations and the results of the sensitivity analysis, combining scale-up and process optimization will most likely drastically reduce the carbon footprint of the process.   Regarding the last comment, we have revised the manuscript, and state clearly at the beginning of the carbon footprint section that the scope is an initial evaluation. We thank the reviewer for the suggestion, and we added it on page 16, lines 632-634.   Page 14 A carbon footprint assessment was carried out based on the described results. Given the small scale at which the microbial oil was produced, this analysis was conducted as an initial evaluation of the process, with the aim to highlight the hotspots for a sustainable scale-up.   Page 16 As expected, the carbon footprint of laboratory scale microbial oil is 28-168 times higher than industrial vegetable oil production, indicating that substantial optimization is required before environmental competitiveness can be achieved.

**Comment: **3. Incomplete Sensitivity Analysis Issue: Only electricity source variation is analyzed. Critical parameters that substantially affect environmental impact are omitted.   Required additions:

- Lipid extraction efficiency (current 35% vs. theoretical 73% achieved in optimization experiments)
- Fermentation yield variations (0.47 vs. 0.73 g_oil/g_CDW)
- Solvent recovery and recycling rates (currently assumed zero recovery, yet solvents contribute 36.0 kgCO₂eq/kg or 51% of scenario 2 impact)
- C/N ratio optimization effects on yield
- Plant capacity scenarios (batch size effects)
- Present results showing relative impact of each parameter on the overall carbon footprint to identify which factors most critically affect environmental performance and where optimization efforts should focus.

  RECOMMENDATION Major revisions required to address the three issues above. The fermentation and extraction optimization work is solid, but the LCA component requires substantial methodological corrections to meet scientific standards.

**Response: **We thank the reviewer for the suggestion to include a more detailed sensitivity analysis. Based on the available data, we evaluated the carbon footprint keeping into account the lipid extraction efficiency, the fermentation yield variation and solvent recovery. We included the results in pages 14-16, and in Figure 6.   Plant capacity scenarios were not analyzed due to lack of information; more upscaled data from a TEA would be needed to effectively discuss this aspect. C/N ratio optimization was not considered as no in vivo data is available; however, the oil yields in the screening experiments are close to the highest ones reported for oleaginous yeasts, suggesting that further optimization of this parameter would only bring to very small changes in the yield. We now discuss the fermentation yield more in detail in the manuscript:   Page 15 The higher yield in microtiter plates could be attributed to a plethora of factors, most likely to a condition where the cells were more stressed due to, as an example, a lower pH and a lower %DO, if compared to the bioreactor fermentation. These process parameters could be easily tweaked and optimized in a bioreactor.   Finally, we would like to underline that solvent recovery was indeed already considered, with a recycling rate of 90% (as we measured experimentally). This aspect was most likely not clear, so we clarify it on Page 14. No further optimizations were performed, as 90% recovery was considered to be already a very high percentage.   Sensitivity analysis on the lipid extraction efficiency and fermentation yield variation was conducted and described in Pages 14-16; results are shown in Figure 6.

# Reviewer report: Reviewer response for version 1

**DOI:** 10.21956/openreseurope.22564.r60221

**Published:** 2025-09-27

**Authors:** Wilailuk Niyommaneerat

**Affiliations:** 1 Sustainable Environment Research Institute, Chulalongkorn University, Bangkok, Bangkok, Thailand

*“With the aim of investigating the potential of LE in the production of microbial oil, in this study we focused on the ability of C. oleaginosum to grow and accumulate lipids using this side stream rich in sugars. Moreover, we also proposed an alternative approach for the extraction of lipids from yeast biomass, substituting the traditional potentially carcinogenic Folch protocol (requiring chloroform and methanol) with a combination of two green and safer solvent blends (ASTROBIO™ Green Solvents) chosen from the portfolio of Soft Chemicals S.r.l.”*

 1. The above statement is quite conflict with the topic as focusing on Carbon footprint of microbial oil from waste lemon peel extract and the part of the results showed that carbon footprint is much contributed than conventional vegetable oil. What is the research more focus on environment impact of microbial oil or waste valorization?

 2. In somehow as microbial oil in this study were defined as Green Solvent: please elaborate in this study as the process of LE extract and microbial fermented required solvents and chemicals. Please state in the introduction or summary of the finding.

 3. Please add clarification of the details of function unit as 11.79 g of microbial oil from lemon extract. What application will you for LCI database and further application of this data?

 4. What is the significant finding based on the research otherwise potential added value of agricultural waste. The comparative analysis between conventional waste management and production as value added product (microbial oil).

Is the study design appropriate and does the work have academic merit?

No

Is the work clearly and accurately presented and does it cite the current literature?

Yes

If applicable, is the statistical analysis and its interpretation appropriate?

I cannot comment. A qualified statistician is required.

Are all the source data underlying the results available to ensure full reproducibility?

Partly

Are the conclusions drawn adequately supported by the results?

Yes

Are sufficient details of methods and analysis provided to allow replication by others?

Yes

Reviewer Expertise:

Environmental Management and Sustainability, Municipal Waste Management and Circular, Economy- Enterprise-level, greenhouse gas assessment- Environmental and toxicological risk assessment.

I confirm that I have read this submission and believe that I have an appropriate level of expertise to confirm that it is of an acceptable scientific standard, however I have significant reservations, as outlined above.

# Response

**Published:** 2025-11-27

**Authors:** Vittorio Giorgio Senatore

**Comment: **“With the aim of investigating the potential of LE in the production of microbial oil, in this study we focused on the ability of C. oleaginosum to grow and accumulate lipids using this side stream rich in sugars. Moreover, we also proposed an alternative approach for the extraction of lipids from yeast biomass, substituting the traditional potentially carcinogenic Folch protocol (requiring chloroform and methanol) with a combination of two green and safer solvent blends (ASTROBIO™ Green Solvents) chosen from the portfolio of Soft Chemicals S.r.l.” 1. The above statement is quite conflict with the topic as focusing on Carbon footprint of microbial oil from waste lemon peel extract and the part of the results showed that carbon footprint is much contributed than conventional vegetable oil. What is the research more focus on environment impact of microbial oil or waste valorization?  

**Response:** We agree with the reviewer on the fact that currently this proof-of-concept, lab-scale process is not more sustainable than the industrial production of coconut oil. To answer the last question, the focus of this work was indeed both on waste valorization and environmental impact. The reason we performed a carbon footprint analysis this early in process development was to conduct an initial assessment of the hotspots of the process, highlighting important aspects to keep into consideration when scaling up. We included a disclaimer at the beginning of the carbon footprint section (see below, Page 14).   Page 14 A carbon footprint assessment was carried out based on the described results. Given the small scale at which the microbial oil was produced, this analysis was conducted as an initial evaluation of the process, with the aim to highlight the hotspots for a sustainable scale-up.

**Comment:**  2. In somehow as microbial oil in this study were defined as Green Solvent: please elaborate in this study as the process of LE extract and microbial fermented required solvents and chemicals. Please state in the introduction or summary of the finding.  

**Response: **We believe that a misunderstanding might have led to the following comment. Throughout this work, the term ‘green solvent’ refers to the use of the blend BA/K1 during the extraction process of the microbial oil. The microbial oil obtained is the target product to be extracted from within the yeast cells, not the solvent itself.  

**Comment: **3. Please add clarification of the details of function unit as 11.79 g of microbial oil from lemon extract. What application will you for LCI database and further application of this data?  

**Response: **We agree with the reviewer that the choice of 11.79 g  as functional unit was arbitrary, despite the fact that 11.79 g of oil was the quantity effectively produced at UNIMIB. As suggested by another reviewer as well, we changed the functional unit to 1 kg of microbial oil (ISO 14040/14044) to facilitate the interpretation of the results.   Regarding the comment on the inventory, the LCI data generated in this study are publicly available as “underlying data,” in accordance with the journal’s guidelines. In addition, it enables comparative (lab-scale) LCAs with conventional lipid feedstocks and provides a basis for modeling scale-up scenarios, process optimization strategies, and downstream applications. In future work, this dataset may be further expanded and validated at pilot scale to broaden its applicability.  

**Comment: **4. What is the significant finding based on the research otherwise potential added value of agricultural waste. The comparative analysis between conventional waste management and production as value added product (microbial oil).  

**Response: **We thank the reviewer for the suggestion. We included a comment in the conclusion.   Page 18 Overall, this study demonstrates that lemon peel side-streams can be efficiently upcycled into a fermentable extract, subsequently converted into microbial oil. This highlights the potential of agricultural by-products as feedstock for value-added bioprocesses. In contrast to conventional management practices for lemon peel waste (e.g., low-value feed, composting or incineration), our approach illustrates a circular bioeconomy pathway where waste is transformed into microbial lipids with potential applications in biofuels, bioplastics or cosmetic ingredients.

# Reviewer report: Reviewer response for version 1

**DOI:** 10.21956/openreseurope.22564.r60222

**Published:** 2025-09-16

**Authors:** Luis Alfonso Díaz-Secades

**Affiliations:** 1 Universidad de Oviedo, Oviedo, Spain

This study develops a fermentation process to produce microbial oil from lemon peel extract, an agro-industrial waste stream, and evaluates its environmental performance through carbon footprint assessment. The results show promising lipid yields and highlight that scaling up and solvent optimization are essential for making the process more sustainable and competitive with conventional vegetable oils.

**Introduction**

- The introduction cites recent literature but could better highlight the gap this study fills.
- Why was lemon peel chosen over other citrus or agro-wastes? If provide data on global lemon waste quantities to justify relevance.
- The rationale for choosing *C. oleaginosum* is given, but comparison with other oleaginous yeasts seems to be a bit limited.

**Materials and Methods**

- Statistical analysis methods are not explained in detail, in particular the significance testing.
- Why was urea chosen over other nitrogen sources besides pH control?
- Please, provide justification for the chosen LCA boundary and if possible add sensitivity analysis for LCA assumptions.

**Results and Discussion**

- I believe the **c**omparison with other studies is limited to carbon footprint.
- The discussion does not integrate techno-economic feasibility with sustainability.

**Conclusions**

 How could solvent use be reduced or recycled?

Is the study design appropriate and does the work have academic merit?

Yes

Is the work clearly and accurately presented and does it cite the current literature?

Partly

If applicable, is the statistical analysis and its interpretation appropriate?

Partly

Are all the source data underlying the results available to ensure full reproducibility?

Yes

Are the conclusions drawn adequately supported by the results?

Partly

Are sufficient details of methods and analysis provided to allow replication by others?

Partly

Reviewer Expertise:

Carbon footprint analysis.

I confirm that I have read this submission and believe that I have an appropriate level of expertise to confirm that it is of an acceptable scientific standard, however I have significant reservations, as outlined above.

# Response

**Published:** 2025-11-27

**Authors:** Vittorio Giorgio Senatore

**Comment: **This study develops a fermentation process to produce microbial oil from lemon peel extract, an agro-industrial waste stream, and evaluates its environmental performance through carbon footprint assessment. The results show promising lipid yields and highlight that scaling up and solvent optimization are essential for making the process more sustainable and competitive with conventional vegetable oils.   Introduction The introduction cites recent literature but could better highlight the gap this study fills. Why was lemon peel chosen over other citrus or agro-wastes? If provide data on global lemon waste quantities to justify relevance. The rationale for choosing C. oleaginosum is given, but comparison with other oleaginous yeasts seems to be a bit limited.  

**Response: **We thank the reviewer for the suggestions. For both questions, we need to point out that this paper is the result of a European Horizon 2020 project, and poses itself as a case study and proof of concept of sustainable upcycling of agricultural waste, rather than a systematic study.   In terms of the choice of substrate, we included the following in the introduction (Page 3): Globally, lemon production exceeds 20 million tonnes per year (FAO, 2021), and industrial processing produces large volumes of by-products, with peels representing the major fraction, accounting for approximately 40–50% of the fruit weight (Negrea et al., 2025).   Regarding the choice of the yeast, as anticipated above, we selected C. oleaginosum as a proof of concept. We changed the text to make it more evident, while also mentioning other industrially-relevant oleaginous yeasts (Page 3).  

**Comment: **Materials and Methods Statistical analysis methods are not explained in detail, in particular the significance testing. Why was urea chosen over other nitrogen sources besides pH control? Please, provide justification for the chosen LCA boundary and if possible add sensitivity analysis for LCA assumptions.  

**Response: **We thank the reviewer for spotting the mistake about the statistical analysis: we included a dedicated paragraph at the end of Materials and Methods section (Page 10):   Statistical analysis GraphPad PRISM 10.1.0 was used for the statistical analysis of fermentation parameters. For the medium optimization experiment, a two-way ANOVA was performed to analyse differences between samples, followed by a post hoc Tukey–Kramer test for multiple comparisons. The remaining statistical analyses were performed using a two-tailed, unpaired, heteroscedastic Student’s t-test.   As far as urea is concerned, we already state in the text why we chose this nitrogen source over the more commonly used ammonia. We have colored the sentence in blue in the main text and we reported it below (Page 12): Urea was chosen over ammonium sulfate as it has been shown that in batch fermentations the latter causes a significant drop in the pH during growth (Mastella et al., 2022).   Sensitivity analysis was conducted and added to the manuscript. The carbon footprint calculation’s system boundary (cradle-to gate) was selected because data was not available on the downstream use, transport and end-of-life life cycle stages. This justification is added to page 8. For the lemon burden assumption, text about the small contribution of the lemon production to the carbon footprint of a wider A2C food waste system was added to p. 9 to justify the assumption. Regarding the resin production assumption, it was added to the supplementary data (Section 2 - Assumptions - Assumptions in the life cycle stages - Lemon extract manufacturing - Purification) that in the A2C project the burden from reusable resins was divided to other products besides the microbial oil.  

**Comment: **Results and Discussion I believe the comparison with other studies is limited to carbon footprint. The discussion does not integrate techno-economic feasibility with sustainability.  

**Response: **We thank the reviewer for the suggestions, and we do agree that a techno-economic feasibility study should be conducted, however we decided not to include it because of the very limited available literature, as well as the fact that such analysis was outside of the scope of the Agro2Circular European project. As this point is relevant, we included a comment in the discussion (page 16):   To evaluate the carbon footprint of the process in a more robust manner, a scale-up to the industrial level of the microbial oil production system and downstream procedures is required to gather precise information on production processes, electricity consumption, and chemical emissions. Also, a techno-economic assessment should be performed to evaluate the technical and economic feasibility of microbial oil production.  

**Comment: **Conclusions How could solvent use be reduced or recycled?  

**Response: **We thank the referee for the interesting question. Solvents are currently recycled (90% recovery); as we realized this was not clear, we specified it in page 14, line 566. In general, we believe that solvent use could be reduced by improving both the extraction strategy (e.g., a soxhlet extraction just to name one), and the yield of the process. Indeed, less solvent would probably be needed to extract a higher amount of oils. We do agree that an optimization of the extraction method should be carried out. We added a few comments in this regard in the new section on the sensitivity analysis and in the conclusions.    Page 15 Regarding the solvent extraction, the strategy proposed in this paper for large scale extraction is very simple and rudimentary. For the sensitivity analysis, we selected a 90% extraction efficiency of the blend BA/K1 compared to the traditional MeOH/Chloroform method: the value is only slightly higher than the one measured experimentally, and most likely will be improved with a more sophisticated extraction set up, like with a soxhlet extractor.    Page 16 The carbon footprint of the chemicals and solvents was 36.0 kgCO _2_eq/kg microbial oil. This is significantly higher than all the benchmarks listed by Masri et al. (2019) and Abelleira et al. (2024) and more than 14 times higher than the carbon footprint of coconut oil. Therefore, it is essential to reduce chemical and solvent emissions through efficient recycling of chemicals, improving extraction methods as shown in the sensitivity analysis, and, most importantly, by improving the yield through precise monitoring of the fermentation parameters.   Page 17 Green solvents used in this study, which are bio-based blends of small esters and alcohols, could be efficiently separated from the oils and thus efficiently recycled with several extraction techniques like soxhlet extraction, standard or extractive distillation as well as less energy intensive procedures like membrane-based pervaporation fostering overall sustainability and financial viability and of the process.
